# Piezoelectric Active Sensing-Based Pipeline Corrosion Monitoring Using Singular Spectrum Analysis

**DOI:** 10.3390/s24134192

**Published:** 2024-06-27

**Authors:** Dan Yang, Hu Wang, Tao Wang, Guangtao Lu

**Affiliations:** 1 Key Laboratory for Metallurgical Equipment and Control of Ministry of Education, Wuhan University of Science and Technology, Wuhan 430081, Chinaluguangtao@wust.edu.cn (G.L.); 2 Hubei Key Laboratory of Mechanical Transmission and Manufacturing Engineering, Wuhan University of Science and Technology, Wuhan 430081, China; whcomeon2022@163.com; 3 Precision Manufacturing Institute, Wuhan University of Science and Technology, Wuhan 430081, China

**Keywords:** corrosion of pipeline, piezoelectric transducers, active sensing, singular value analysis

## Abstract

Pipelines are an important transportation form in industry. However, pipeline corrosion, particularly that occurring internally, poses a significant threat to safe operations. To detect the internal corrosion of a pipeline, a method utilizing piezoelectric sensors alongside singular spectrum analysis is proposed. Two piezoelectric patches are affixed to the exterior surface of the pipeline, serving the roles of an actuator and a sensor, respectively. During the detection, the signals excited by the actuator are transmitted through the pipeline’s wall and are received by PZT2 through different paths, and the corresponding piezoelectric sensor captures the signals. Then, the response signals are denoised by singular spectrum analysis, and the first several wave packets in the response signals are selected to establish a feature for pipeline corrosion detection. At last, the envelope area of the selected packets is calculated as a feature to detect corrosion. To validate the proposed method, corrosion monitoring experiments are performed. The experimental results indicate that the envelope area of the first several wave packets from the response signals, following singular spectrum analysis, can serve as a feature to assess the degree of pipeline corrosion, and the index has a monotonic relationship with the corrosion depth of the pipeline. This method provides an effective way for pipeline corrosion monitoring.

## 1. Introduction

Pipelines are an important way to transport fluids in the field of energy [1,2,3]. Corrosion is one of the most hazardous damaging mechanisms in a pipeline [4,5], especially scouring corrosion of the inner walls of pipelines. If not detected and repaired in time, the structural integrity of the pipeline will be compromised in addition to potential leakage and pollution. According to the World Corrosion Organization [6], the damage cost caused by corrosion is around USD 2.5 trillion globally, and therefore, the detection of pipeline corrosion is of great significance [7] to ensure its safe operation.

Pipeline corrosion monitoring has received much attention [8,9,10]. Commonly used methods for pipeline corrosion inspection include visual inspection, magnetic particle inspection, radiographic testing [11], and eddy current testing [12], among others. Circumferential waves propagate in the circumferential direction and are used for detecting pipe corrosion and other defects since they are very sensitive to small defects in pipe wall thickness. Da Y et al. [13] develop a technique to accurately reconstruct pipeline defects by investigating the theoretical dispersion equations of circumferential guided waves. Lyu F et al. [14] investigate the application of ultrasonic guided wave technology in the detection of corrosion defects in pipelines. Rodgers et al. [15] propose a novel guided wave inspection technique that can be used to detect axial cracks in pipelines. Shi W et al. [16] provide a method for detecting the circumferential crack by analyzing the two reflected SH0 guided wave from the front edge and back edge of the circumferential crack. However, the current study does not give a quantitative relationship between the ultrasonic guided wave signal and the degree of corrosion. Additionally, these methods are typically implemented at fixed intervals and cannot provide real-time monitoring of pipelines. However, during the inspection interval, some severe pipeline corrosion may occur, causing significant losses and hazards, which is not feasible for pipelines that need to operate continuously. Therefore, it is necessary to find a method for real-time monitoring of internal corrosion in pipelines.

In comparison to the above techniques, the approach of structural health monitoring using piezoelectric active sensing provides real-time monitoring of structures [17,18]. Piezoelectric patches are often surface-bonded on or embedded in a structure, with one of them acting as an actuator to generate excitation, and the other one receiving the response signal as a sensors [13,19,20,21]. Then, by comparing the changes in the response signals before and after structural damage, structural damage can be identified, thus providing a basis for real-time monitoring of structural health status [22,23]. Siswantoro et al. [24] verified the possibility of piezoelectric sensors in pipeline monitoring. Yu et al. [25] had embedded multiple Lead Zirconate Titanate (PZT) sensors at different positions along a pipeline to achieve real-time monitoring of parameters such as strain, temperature, and pressure in the pipeline. A microcontroller was used to collect and process the signals picked by the sensors. This methodology can detect defects and cracks accurately in the pipeline and provide early warning before a pipeline failure occurs.

Moreover, the pipelines are sometimes located in high-noise environments, which can cause interference and disrupt the features in the response signals obtained by the piezoelectric sensors. Traditional noise reduction methods typically rely on time or frequency domain signal processing techniques, such as filters and wavelet transforms, etc. [26,27,28]. They work well for signals with low levels of noise; however, their effectiveness becomes unstable when dealing with nonlinear or non-stationary signals. In addition, Singular Spectrum Analysis (SSA) performs better in denoising nonlinear and non-stationary signals compared with the noise reduction algorithms as mentioned heretofore. SSA is a robust technique for analyzing time series data [29]. SSA creates a trajectory matrix derived from the time series data under study and then proceeds to decompose and subsequently reconstruct this matrix, aiming to distill distinct signals that correspond to various elements of the original time series. These elements may include sustained trend signals, cyclical patterns, as well as random noise components.

SSA can effectively reduce noise and interference in a signal while preserving the main features of the signal. Luong et al. [30] incorporated a multivariate singular spectral analysis (MSSA) with the electromagnetic testing systems to improve the signal-to-noise ratio in the inspection of the outer surface corrosion in a moisture separator reheater tube. This analytical approach facilitated the extraction of the corrosion-related fluctuations embedded within the signals. Li et al. [31] proposed a method that combines SSA with Long Short-Term Memory (LSTM) networks to detect corrosion spots on underwater oil pipelines. The method adopted SSA to decompose time series data into a set of components, which were then fed into an LSTM network for training and prediction of the corroded areas of the pipeline, which was instrumental in facilitating the digital management of corrosion risks associated with underwater operations.

In this paper, a piezoelectric active sensing method with SSA is proposed for monitoring pipeline internal corrosion. SSA is used to reduce the noise of the acquired response signal. Two piezoelectric patches are bonded on the outer wall of the pipeline as an actuator and a sensor, respectively. The piezoelectric actuator generates an excitation and the piezoelectric sensor is used to receive the ultrasonic signal propagating through the wall of the pipe. For the data processing, the response signal is first decomposed into multiple signals. According to the obtained eigenvalues and their corresponding coefficients, the preceding signals are selected for reconstruction to remove noise. Then, the envelope area of the first several wave packages is chosen as the damage index (DI) to characterize the internal corrosion degree. An experimental apparatus involving a pipe segment is built to verify the proposal method.

The rest of the paper is organized as follows: Section 2 introduces the theoretical fundamentals and proposes the monitoring principle. Section 3 presents the experimental setup to verify the proposed method. Section 4 discusses the experimental results. Section 5 concludes the paper.

## 2. Theoretical Fundamentals

### 2.1. Working Principle

**Figure 1:**
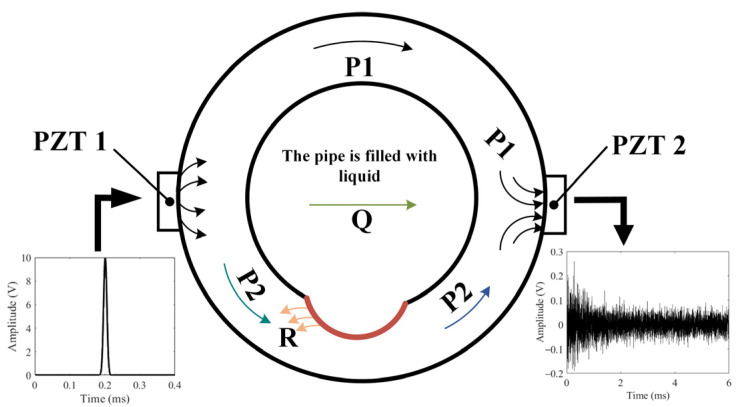
Schematic diagram (the propagation path of waves in a crosssection of the pipeline).

The operational mechanism of the active sensing technique using two piezoelectric transducers for detecting pipeline corrosion is depicted in Figure 1.

A pair of PZT patches are bonded to the pipeline’s exterior surface. One, designated as PZT1, functions as the actuator to produce excitation, while the other, known as PZT2, serves as the sensor to detect the signals. Ultrasonic wave is generated by applying a Gaussian pulse voltage signal to PZT1. As shown in Figure 1, the ultrasonic wave generated by PZT1 travels in different ways in the pipeline wall and inner liquid. The ultrasonic waves generated by PZT1 propagate to PZT2, which acts as a sensor, through three pathways and are then captured by PZT2: A portion of the ultrasonic waves propagate in the upper part of the pipe wall. Since this part of the pipe wall is not corroded, the propagation characteristics of these ultrasonic waves remain unchanged. This is marked as P1; a portion of the ultrasonic waves propagate through the inner pipe wall interface and then into the liquid inside the pipe, finally reaching PZT2. This portion is marked as Q. Since the liquid inside the pipe remains basically unchanged, the propagation characteristics of these ultrasonic waves also remain relatively stable. Another portion propagates in the lower part of the pipe wall to PZT2, marked as P2. Since this part of the pipe wall is corroded, the propagation characteristics of the ultrasonic waves in this region will change. From the above analysis, it can be seen that the received ultrasonic waves are primarily composed of these three parts. Since only the propagation path of P2 has changed, the characteristic in the received signals is mainly caused by the corrosion.

Since the signals have different propagation paths, the arrival time at the PZT2 is also different. Q (through liquid) and R (through reflection and refraction in corroded regions), which take longer to reach the sensor, can be separated by analyzing the first few wave packets of the received signals by means of the leading wave. And P1 (through the uncorroded region) is almost constant; therefore, the method proposed in this paper mainly analyzes P2 (through the corroded region). As the corroded region deepens, the reflection and refraction waves R occurring in the corroded region increase, which leads to the decrease of P2, and the total signal received by PZT2 decreases. Therefore, using the first few wave packets received by PZT2, this study employs the enveloping area of leading waves as the defect feature to represent the degree of corrosion.

### 2.2. Signal Analyzing and Processing

**Figure 2:**
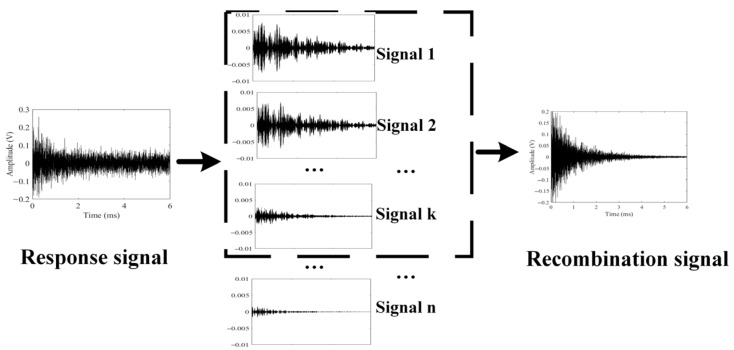
The returned signal undergoes SSA decomposition to obtain the recombination signal.

Signals collected by PZT2 are easily affected by environmental noise, and the key information of the signal cannot be directly analyzed. After singular spectrum analysis (SSA), the signal is decomposed into multiple one-dimensional signals. As shown in Figure 2, the response signal was decomposed into several one-dimensional signals. The amplitudes of the first few signals are approximately 0.01 V, while the amplitudes of the last few signals are close to 0 V. By selecting the first few sets of data for reconstruction, noise interference can be reduced, resulting in a signal that characterizes pipeline corrosion. The response signal is decomposed and reconstructed after SSA, forming a new signal. The details of SSA will be introduced in the next section.

### 2.3. Singular Spectrum Analysis

Based on singular value decomposition (SVD), singular spectrum analysis is an effective time series analysis algorithm that can decompose a signal into a batch of summable orthogonal time series [32]. These time series represent the trend component, periodic component, and noise component of the original signal. Therefore, singular spectrum analysis is adopted to reconstruct trend items and extract features of the noise-contaminated response signal in the proposed method. The detailed procedures of the SSA are illustrated below [33]:

Step 1: Time series decomposition embedding to form a trajectory matrix

The response signal is actually a nonzero time series. The Hankel matrix of the original response signal is obtained by using the delay embedding technique, and the obtained Hankel matrix is given as follows, which means that all the elements along the diagonal *i* + *j* = const are equal:

(1) $$X=\left[X_{1}{:\ldots \;:X}_{K}\right]={\left(x_{ij}\right)}_{i,j=1}^{L,K}=\left(\begin{array}{ccc} y_{1} & \cdots & y_{K}\\ \vdots & \ddots & \vdots \\ y_{L} & \cdots & y_{N} \end{array}\right)$$

where K = T − L + 1, L ≤ T/2, and 2 ≤ L ≤ T.

From the perspective of column vectors, X can be rewritten as follows:

(2) $$X=\left[y_{1}{,y_{2},\ldots ,:y}_{K}\right]$$

(3) $$y_{i}={\left[y_{i},y_{i+1},\cdots {,y}_{i+L-1}\right]}^{T}$$

And $y_{i}$ consists of a subsequence of length *L* from **y**_2_ with a stride of 1, starting from the first element. This is equivalent to sliding a window of length *L* from left to right, extracting the elements covered by the window to form a subsequence, which then serves as the column vectors of matrix X.

Step 2: Singular value decomposition

The trajectory matrix X undergoes the process of Singular Value Decomposition. Let $S=XX^{T}$,${\;\lambda }_{i}(i=1,\ldots ,L)$ be the eigenvalue of S, and $\lambda_{1}\geq \lambda_{2}\geq \ldots \geq \lambda_{L}\geq 0$. The orthonormal vector of the matrix S corresponding to these eigenvalues is $U_{i}(i=1,2,\ldots ,L)$. The corresponding eigenvectors $V_{i}=X^{T}\left(U_{i}/\sqrt{\lambda_{i}}\right)$, where i=1,2,⋯,r, and *r* is the rank of the matrix X and the number of nonzero singular values. $\sqrt{\lambda_{i}}$ represents a singular value. In this case, the SVD of the trajectory matrix X is expressed as follows:

(4) $$X=\sum_{i=1}^{r}{\sqrt{\lambda_{i}}U}_{i}V_{i}^{T}=X_{1}+X_{2}+\cdots +X_{r}$$

Step 3: Grouping

The objective of categorization is to transform the matrix X into submatrices that are linearly independent:

(5) $$X=X_{1}+X_{2}+\cdots +X_{m}$$

The detailed procedure is outlined below: Initially, the singular values are categorized into m clusters, represented as $\left\{I_{1},I_{2},\ldots ,I_{m}\right\}.$ For example, if $I_{1}=\left\{\sigma_{1},\;\right.\left.\sigma_{3}\right\},\;I_{2}=\left\{\sigma_{2},\;\right.\left.\sigma_{4},{\;\sigma }_{6}\right\}$. For each cluster, the associated submatrix is constructed by multiplying the respective singular value with the corresponding left and right singular vectors. For example, for group *I*_1_, its corresponding submatrix is as follows:

(6) $$X_{1}=\sigma_{1}U_{1}V_{1}^{T}+\sigma_{3}U_{3}V_{3}^{T}$$

And each nonzero singular value is, by default, treated as a separate group, resulting in the grouped Formula (3), which represents truncated singular value decomposition.

Step 4: Anti-diagonal average

After the diagonal averaging algorithm, which is a linear transformation method, each matrix $X_{I_{j}}$ obtained by SVD is converted into a new series of length N, and the decomposed sequence is obtained. Let Y be a L×K matrix with elements $y_{ij}$, where 1≤i≤L and 1≤j≤K. We set $L^{*}=\min(L,K)$,${\;K}^{*}=\max(L,K)$ and N=L+K−1. If L<K, $y_{ij}^{*}=y_{ij}$, Otherwise, $y_{ij}^{*}=y_{ji}$, Then the matrix Y can be transformed into a series $y_{1},y_{2},\cdots ,y_{N}$ obtained by the formula as follows [34]:

(7) $$y_{k}=\left\{\begin{array}{ll} \frac{1}{k}\sum_{t=1}^{k}y_{t,k-t+1}^{*} & 1\leq k\leq L^{*}\\ \frac{1}{L^{*}}\sum_{t=1}^{L^{*}}y_{t,k-t+1}^{*} & L^{*}\leq k\leq K^{*}\\ \frac{1}{N-K+1}\sum_{t=k-K^{*}+1}^{N-K^{*}+1}y_{t,k-t+1}^{*} & K^{*}\leq k\leq N \end{array}\right.$$

A two-dimensional matrix can be converted into a one-dimensional matrix by Formula (3). Therefore, the response signal can be transformed into multiple sets of one-dimensional sequences. Arrange one-dimensional sequences in descending order of eigenvalues Then, the vectors corresponding to the first *n* eigenvalues are selected to reconstruct a new matrix. Experimental Setup and Procedure.

## 3. Experimental Setup and Procedure

### 3.1. Experimental Device and Test Specimen

**Figure 3:**
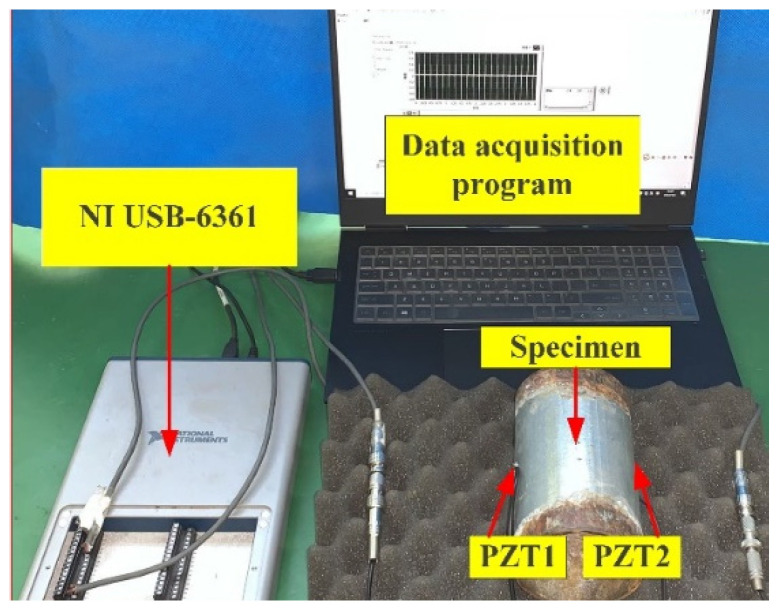
Experimental setup.

In accordance with the aforementioned concepts, the experimental setup is shown in Figure 3. This setup includes a test pipeline segment with a pair of PZT patches attached to its outer surface, a multifunction acquisition device (NI USB-6361, National Instruments, Austin, TX, USA), and a laptop computer with a data acquisition program developed in LabVIEW.

As mentioned above, two PZT patches are bonded on the outer surface of the sample pipeline segment in an axial horizontal position, as shown in Figure 2. PZT1 generates an excitation signal that propagates to PZT2, and PZT2 is connected to the data acquisition unit for gathering the response signals.

**Figure 4:**
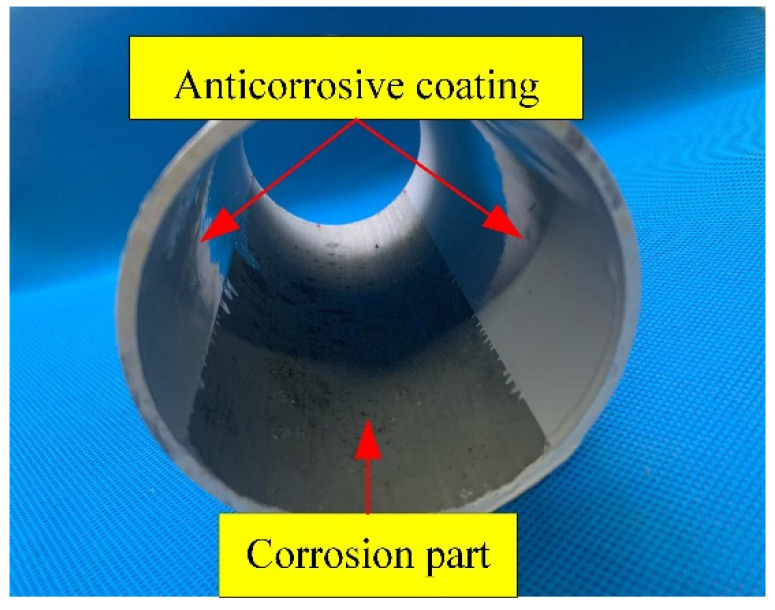
Corrosion area of sample pipeline.

As shown in Figure 4, the Q235 steel specimen pipeline segment is 200 mm long with 88 mm outside diameter and 82 mm inside diameter. Table 1 displays the material of the pipeline segment. During the experiment, the corrosion area in the inner wall of the pipeline should be strictly controlled to maintain experimental consistency. Therefore, the non-corrosion area inside the pipeline is painted with an insulation protection layer. Temperature and liquid level changes will have an effect on transducer coupling and propagating waveform [35,36]. In the experiments, the conditions were strictly controlled at room temperature, and the same liquid level was maintained.

**Figure 5:**
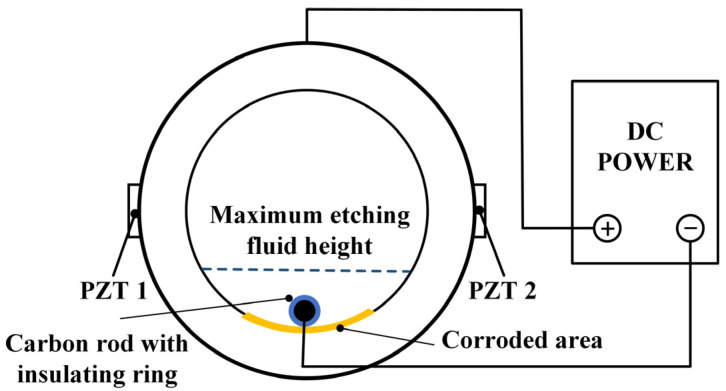
Electrochemical corrosion.

Electrochemical corrosion is used to simulate the corrosion process of pipeline inner walls [37], as shown in Figure 5. A graphite rod, 6 mm in diameter and 100 mm in length, serving as the negative electrode in the electrochemical corrosion process [38], is attached to the negative port of an external DC power source (Delixi Electric Ltd., Yueqing, China). The pipeline serves as the anode for electrochemical corrosion and is connected to the positive terminal of the external DC power supply. The electrochemical DC power supply provides a constant output voltage of 12 V and an output current of 3 A to ensure the stability of the pipeline corrosion process. The electrolyte solution in the pipeline is a 10% sodium chloride solution with a volume of 400 mL. The graphite rod is placed along the axial direction in the pipeline, and two insulating rings with an inner diameter of 6 mm and an outer diameter of 18 mm support the graphite rod to prevent short circuits between electrodes and ensure the distance of 6 mm between the graphite rod and the pipe wall.

### 3.2. Experimental Procedure

To simulate the different inner wall corrosion statuses of the pipeline, electrochemical corrosion experiments were conducted on the pipeline segment. In the corrosion interval of each 15 h, the piezoelectric active sensing test was performed. Starting from a healthy state (with no corrosion) to 90 h of corrosion, test data were collected for 7 different corrosion states. The experiment was repeated for three pipeline segments of the same specification. In the experiments, the signal chosen for excitation purposes is one that is sinusoidally shaped and modulated by a Gaussian function [39]. A computer-based LabVIEW application is utilized to produce this pulse signal. In order to ascertain the most effective frequency for excitation, a sweeping frequency test is conducted on pipelines. It is found that the response signal energy concentration is around 150 kHz, which is taken as the central frequency of the Gaussian pulse for the excitation signal, and the voltage amplitude is set as 10 V. The parameters of the excitation Gaussian pulse signal are shown in Table 2.

The pulse signal is converted into an analog signal by a multifunctional acquisition device (NIUSB-6361) to activate PZT1. Then, the response signal is sensed by PZT2 and acquired by the acquisition device with a sample frequency of 1 Mhz.

**Figure 6:**
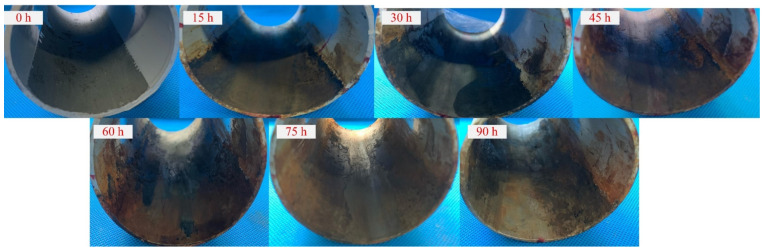
Corrosion status of the pipeline with different corrosion time.

**Figure 7:**
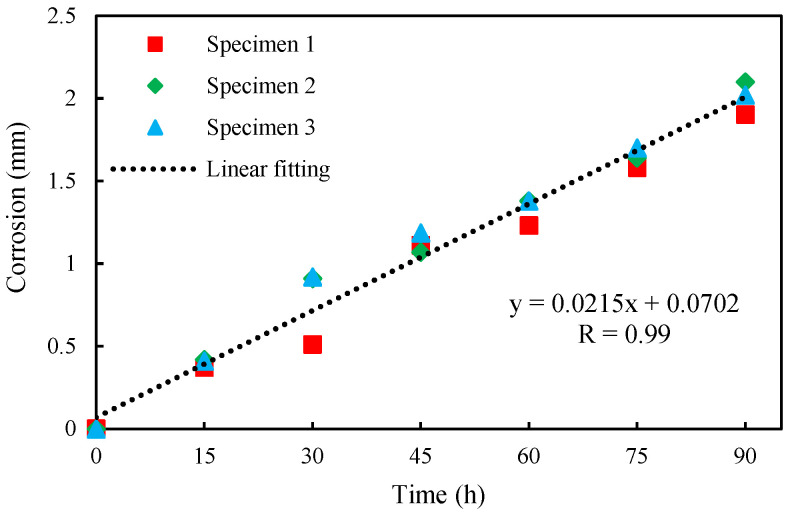
The relationship between the corrosion depth and time.

During the corrosion interval, oxides generated on the inner wall of the pipeline are cleaned for the corrosion depth measuring. Figure 6 shows the pipeline with different corrosion times, i.e., different corrosion depths. The inside diameter of the pipe was measured with an accuracy of 0.01 mm by using an inside diameter gauge 323-134-75 (Japanese SANLIANG, Tokyo, Japanese), and five positions on the inner wall of the pipeline were selected for multiple measurements before taking the average value to obtain the corrosion depth. The relationship between corrosion depth and corrosion time is shown in Figure 7. It shows that there exists a good linear relation between the corrosion depth and the corrosion time. Due to small variations in the experimental conditions and sample pipelines, there are slight fluctuations in the data.

## 4. Experimental Results and Discussion

### 4.1. Signal Processing for Noise Reduction

**Figure 8:**
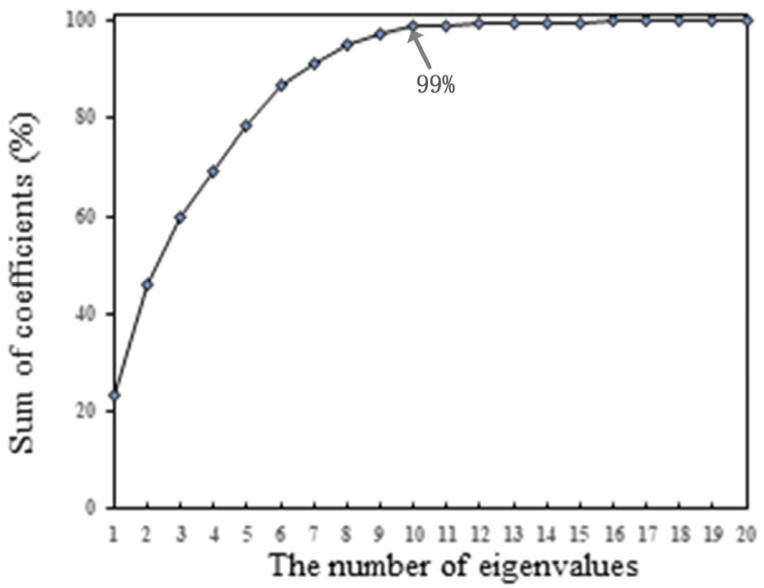
The first i eigenvalues and the sum of corresponding coefficients.

During the data processing, singular spectrum analysis is adopted to decompose and reconstruct the signal to reduce the interference of environmental noise for effective extraction. The embedding dimension of SSA is selected as 20, and the step size is set to 1. During the processing, to obtain the optimal embedding size in SSA, the coefficients of the first few eigenvalues are added to obtain the relationship between the eigenvalues and the sum of coefficients. As shown in Figure 8, the sum of the coefficients corresponding to the first 10 eigenvalues explains 99% of the total sum of coefficients corresponding to all eigenvalues, indicating that the first ten eigenvalues contain the main features of the signal. Therefore, we select the first 10 decomposed signals to reconstruct the signal [40].

### 4.2. Selection of Signals

After denoising the received ultrasonic signal, as described in Section 2, new denoised one-dimensional time signals are obtained. The first few wave packets of the response signal are propagated through the shortest path, excluding reflections and other waves, and are most directly related to the wall thickness in the corroded area. As the propagation time increases, subsequent reflected waves and other refracted waves within the pipe wall gradually arrive. These delayed signals are not as directly related to the wall thickness in the corroded area. Therefore, the envelope area of the first few wave packets is selected to characterize the degree of corrosion in the pipeline. As corrosion intensifies and the wall thickness reduces, the corroded area increases, making the signal propagation more complex, and as the number of reflected waves increases and the number of waves directly passing through *P*2 decreases, using the leading wave packets and ignoring the influence of the reflected waves *R*, the energy of the received waves correlates with the wall thickness in the corroded area. On the other hand, the later wave packets in the signal may contain multiple refractions and reflections on the pipe wall, containing excessive overlapping information. As the propagation time increases, more reflected delayed signals will be received, and the paths of these signals become more complex, but they are not as directly related to the wall thickness of the corroded area. Therefore, it is more appropriate to select the first few wave packets that better reflect the wall thickness to establish a damage index.

**Figure 9:**
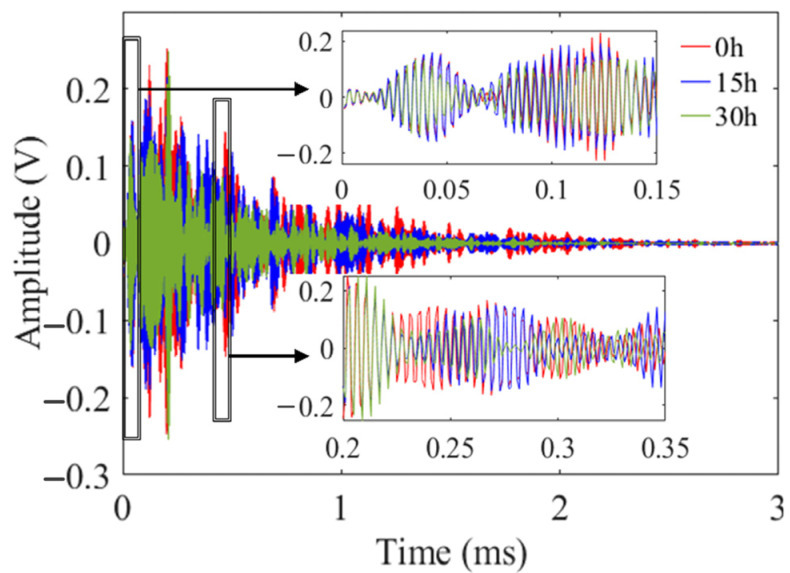
Comparison of response signals of several groups of corrosion acquisition.

The typical response signals of three different corrosion statuses are shown in Figure 9. In order to display the changes in the signal more clearly, the signals of the first and the back few wave packets are detailed displayed. The first few wave packets represent direct responses from the corroded area without interference from subsequent reflected waves. As a result, these wave packets are more sensitive to variations in the depth of corrosion. Therefore, choosing the first few wave packets for analysis can improve the monitoring accuracy for pipeline corrosion.

### 4.3. Damage Index

The envelope area of the first few wave packets after the Hilbert transform is calculated [41,42], and the damage index (DI) to evaluate the pipeline corrosion status is defined as follows:

(8) $$\mathrm{DI}=\int_{a}^{b}Bdt$$

where the variables *a* and *b* denote the initial and final time boundaries of the chosen waveform segments, whereas B corresponds to the voltage amplitude of the respective signal after the Hilbert transform.

### 4.4. Experimental Results

**Figure 10:**
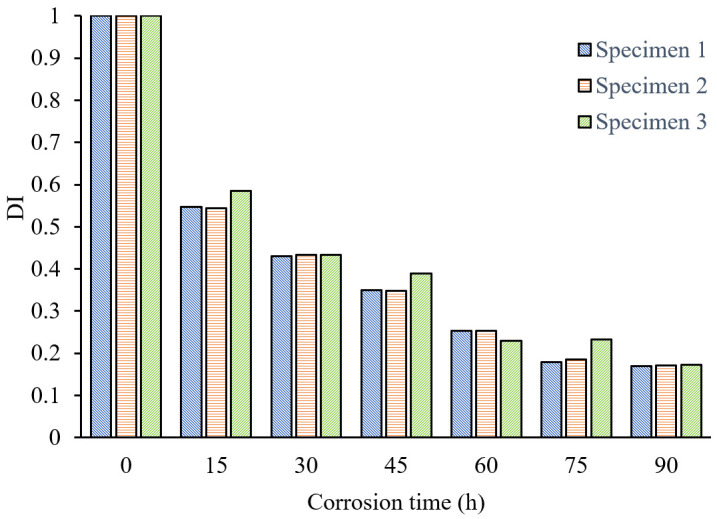
Relationship between DI and corrosion time (signal processed by SSA).

For various stages of pipeline corrosion, the signal’s selected wave packets’ envelope areas are computed following the SSA decomposition process. In order to compare the experimental results of different samples, the DI is normalized. And the DI is defined as 1 for the health state. The relationship between the normalized DI and corrosion time is obtained as shown in Figure 10. The findings from the trials conducted on three pipeline samples suggest that the degree of impedance index (DI) diminishes progressively as the duration of corrosion extends. The DI of the three specimens has good consistency, indicating that the proposed DI can effectively evaluate the degree of pipeline corrosion, especially in the early stage of corrosion (0–15 h), where the DI changes significantly, with an approximate change of 45%. This suggests that the proposed method exhibits strong sensitivity to early-stage corrosion and can be used as an effective tool for monitoring the early corrosion of pipelines.

**Figure 11:**
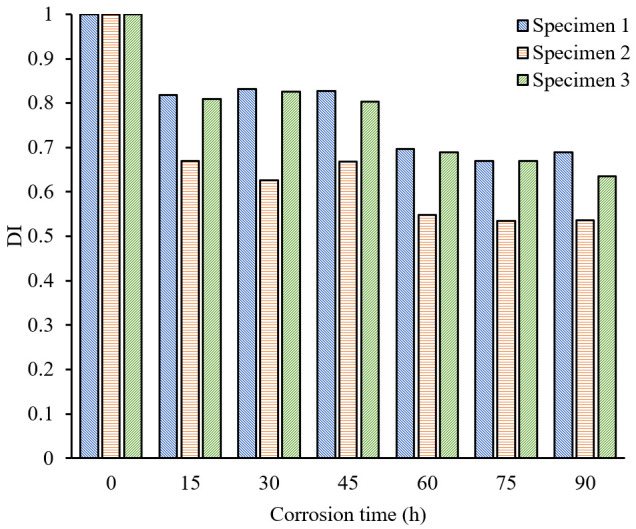
Relationship between corrosion time and DI (signal processed by filtering).

In order to demonstrate the validity of the method, the response signal is denoised with a traditional filter. Then, the denoised signal is used to extract the feature using the same process as described above. The relationship between the DI and corrosion time is shown in Figure 11. It is observable from the illustration that throughout the entire process of corrosion, there is a decline in the value of the DI. However, it does not exhibit a strictly decreasing monotonic relationship with corrosion time. Especially during the time periods of 15–45 h and 60–90 h, there is little observable change in the DI over time. By comparing Figure 10 and Figure 11 in the first 0–30 h, the DI value extracted from the SSA processed signals changes 56.8%, while the DI value extracted from the traditional filtered signals only changes 23.9%. It can be concluded that signals processed with SSA can improve the sensitivity of identifying pipeline corrosion and can detect early pipeline corrosion in a timely manner.

In addition, traditional methods analyze all response signals to interrogate pipeline corrosion. However, it is important to note that the features in the first several wave packets of the response signal are more prominent. This can improve the sensitivity of pipeline corrosion monitoring and provide better corrosion features to evaluate the pipeline corrosion status.

## 5. Conclusions

In this paper, a method is proposed for pipeline corrosion detection by using piezoelectric active sensing combined with singular spectrum analysis. To study the characteristic information carried by the response signal, the first few wave packets in the response signal, rather than the entire signal, are chosen for feature extraction by the theoretical analyses, as the former is not affected by excessive subsequent reflected waves and better represents the corrosion state of the pipeline wall. Singular Spectrum Analysis (SSA) is used to decompose the response signal to reduce the noise interference in the signal, and the method can effectively retain the corrosion features in the signal. The envelope area of the wave packet is used as a new damage index for pipeline corrosion. An experimental apparatus is built, and experiments are carried out. Experimental results show that the proposed damage index has a good monotonic relationship with the degree of pipeline corrosion, effectively representing the degree of pipeline corrosion. Compared with the traditional pipeline corrosion detection and inspection method, this method can monitor pipeline corrosion in real time, especially early corrosion.

During the experiment, the sample pipeline segment is relatively simple. The follow-up experiment will consider the research of different materials and different types of pipes. There is still a gap between the simulated environment of laboratory experiments and the external environment. Further investigation is required to determine if the experimental outcomes of the method can be extended to real-world engineering scenarios. The effects on the transducer coupling and propagation waveforms due to changes in temperature or liquid level will also be taken into account.

## Figures and Tables

**Table 1:** The material properties of the specimen.

Parameter	Values	Unit
Density	7.85	g/cm^3^
Elasticity modulus	200~210	GPa
Strength of extension	370~500	MPa
Linear expansion	1.2 × 10^−5^	1/°C

**Table 2:** Parameters of the pulse waveform.

Parameter	Values	Unit
Amplitude	10	V
Center frequency	150	kHz
Normalized bandwidth	0.8	--

## Data Availability

The data that support the findings of this study are available from the corresponding author upon reasonable request. The writers affirm that there are no apparent conflicts of interest, financial or personal, that could potentially bias the findings presented in this publication.
